# Selectin-Targeting Peptide–Glycosaminoglycan Conjugates Modulate Neutrophil–Endothelial Interactions

**DOI:** 10.1007/s12195-018-0555-6

**Published:** 2018-09-17

**Authors:** James R. Wodicka, Vasilios A. Morikis, Tima Dehghani, Scott I. Simon, Alyssa Panitch

**Affiliations:** 1 Weldon School of Biomedical Engineering, Purdue University, West Lafayette, IN 47907 USA; 2 Indiana University School of Medicine, Indianapolis, IN 46202 USA; 3 Department of Biomedical Engineering, University of California-Davis, Davis, CA 95616 USA

**Keywords:** Glycocalyx, Endothelial dysfunction, Glycosaminoglycan, Selectin, Neutrophil

## Abstract

**Introduction:**

The glycocalyx is a layer of glycoproteins, proteoglycans and glycosaminoglycans that coats the luminal surface of most blood vessels. It effectively regulates adhesive interactions between leukocytes in flowing blood and the endothelium, where during inflammation, binding to E- and P-selectins and intercellular adhesion molecule-1 (ICAM-1) promotes cell tethering and arrest under shear flow.

**Methods:**

In this study, we examine the targeting of E-selectin by an engineered peptide moiety bound to a dermatan sulfate backbone. We further investigate this conjugate, denoted as EC-SEAL, by observing its binding to inflamed endothelium, and quantifying its ability to modulate neutrophil–endothelium interactions.

**Results:**

Binding data reveal that EC-SEAL recognizes domains on E-selectin, and to a lesser degree on P- and L-selectin, and ICAM-1. Further, EC-SEAL increases neutrophil rolling velocity, and decreases neutrophil arrest and migration on inflamed human microvascular endothelial cells under physiologically relevant flow conditions.

**Conclusions:**

We conclude that simple targeting strategies can mimic glycocalyx function under inflammatory conditions, effectively reducing neutrophil recruitment.

**Electronic supplementary material:**

The online version of this article (10.1007/s12195-018-0555-6) contains supplementary material, which is available to authorized users.

## Introduction

Leukocytes play a major role in endothelial cell (EC) dysfunction and related cardiovascular diseases.^8^ Upregulation of adhesion molecules that include E-selectin, P-selectin, and intercellular adhesion molecule 1 (ICAM-1) on the EC surface in response to cytokine stimulation leads to increased leukocyte–endothelium interactions. Specifically, polymorphonuclear leukocytes (neutrophils) adhere to activated endothelium and release cytokines and proinflammatory molecules that exacerbate the disease state.^5^,^13^ Following arrest, neutrophils typically undergo diapedesis into the vessel wall where they secrete additional proinflammatory cytokines, causing SMC proliferation and recruiting more inflammatory cells.^3^,^25^ Thus, there has been a significant effort to regulate recruitment and activation of neutrophils at vascular sites of inflammation.

Neutrophils comprise a part of the body’s innate immune system and are the most common leukocyte found in circulating blood. Under normal conditions, there is minimal interaction between neutrophils and receptors on healthy ECs due in part to the presence of the glycocalyx. The EC glycocalyx serves as a major regulator of neutrophil recruitment by providing an interface between circulating neutrophils and P- and E-selectin expressed on the EC surface. The glycocalyx consists of glycoproteins and proteoglycans that present highly negatively charged glycosaminoglycans (GAGs) to the luminal surface.^21^ The mechanism by which the glycocalyx inhibits neutrophil recruitment is ill defined. However, it is thought that the thickness of the glycocalyx can reach several microns into the vessel lumen, exceeding the length of P- and E-selectin and thereby preventing neutrophil interaction with EC selectins. Once the glycocalyx has been denuded in response to inflammatory stimuli such as TNF-*α*, neutrophil recruitment is enhanced.^21^ Neutrophil interaction with ECs has been characterized as a multistep process, initiated by E- and P-selectin mediated leukocyte rolling along the EC surface.^16^,^23^ This action takes place through the interaction with sLe^x^ tetrasacharides expressed on L-selectin, P-selectin glycoprotein ligand-1 (PSGL-1) and glycoproteins, which are constitutively expressed on the neutrophil surface.^15^,^23^ Furthermore, neutrophil rolling on endothelium can cause secondary capture of additional neutrophils from the blood stream through L-selectin/PSGL-1 interactions.^23^ Selectin-mediated rolling leads to neutrophil arrest and shear resistant attachment to ECs. The primary receptors that enable the transition from rolling to arrest are *β*_2_-integrins which are components of macrophage 1 antigen (Mac-1) and lymphocyte function-associated antigen-1 (LFA-1).^23^ Firm adhesion through *β*_2_-integrins is supported by recognition of EC adhesion molecules, including ICAM-1.^23^ Once engaged, LFA-1/ICAM-1 bonds mechanotransduce a flux of calcium that catalyzes F-actin polymerization and subsequent polarization necessary to facilitate neutrophil migration and diapedesis into the vessel wall.^15^,^16^,^23^ Following diapedesis, neutrophils will continue to migrate to their final site of action.^12^ In the process, they will also release certain proinflammatory cytokines that enhance the recruitment of additional inflammatory cell types.^24^

Given that selectins are the primary initiators of leukocyte recruitment on the EC surface and that they are continuously upregulated in chronic inflammation,^1^ blocking their function could prove invaluable for the treatment of inflammation and associated EC dysfunction. Antibodies and small glycomimetic molecules have been utilized to target selectins and prevent leukocyte binding and the subsequent inflammatory response.^4^,^11^,^14^,^16^,^17^,^19^ However, results have been mixed and few of these are currently available for clinical use.^14^,^15^ Drawing inspiration from the protective glycocalyx layer produced by healthy ECs, we developed a selectin-targeting GAG (termed EC-SEAL). This construct consists of multiple selectin-binding peptides attached to a dermatan sulfate (DS) backbone designed to bind selectins and provide a protective layer that functions to prevent leukocyte binding. Previously, EC-SEAL was shown to decrease platelet binding and activation and reduce thrombosis formation in an animal model of DVT.^26^ Current results indicate that EC-SEAL preferentially binds to E- and P-selectin while exhibiting a weaker interaction with ICAM-1. Neutrophil binding and subsequent diapedesis are reduced in the presence of EC-SEAL. Under physiologically relevant shear conditions, EC-SEAL was found to reduce integrin activation and the transition from rolling to arrest on ECs. These results indicate that EC-SEAL has potential as a treatment to mitigate the inflammatory response observed in endothelial dysfunction.

## Materials and Methods

### EC-SEAL Synthesis

The glycocalyx mimetic (EC-SEAL) was fabricated as previously described.^26^ Briefly, vicinal diol groups on DS (*MW*_avg_ 46,275 Da, Celsus Laboratories, Cincinnati, OH) were oxidized to aldehydes using sodium meta-periodate (Thermo Fisher Scientific, Waltham, MA) in 0.1 M acetate buffer. Oxidized DS was then collected *via* size exclusion chromatography (SEC) and reacted with fluorescent CF633 hydrazide dye (Biotium, Fremont, CA) and an excess of *N*-[*β*-maleimidopropionic acid] hydrazide, trifluoroacetic acid salt (BMPH, Thermo Fisher Scientific), in 1 × phosphate buffered saline (PBS, 1.05 mM KH_2_PO_4_, 155.17 mM NaCl, 2.97 Na_2_HPO_4_–7H_2_O; pH 7.4, Thermo Fisher Scientific). This conjugated the hydrazides on CF633 and BMPH to the aldehyde groups on the oxidized DS. Next, biotinylated peptide was added at a 1:1 molar ratio of biotinylated peptide:DS and an excess of the selectin-binding peptide IELLQARGC (IEL, Genscript, Piscataway, NJ) was conjugated *via* the cysteine thiol to the maleimide groups on DS–BMPH in 1 × PBS. Semicarbazide hydrochloride (Sigma-Aldrich, St. Louis, MO) was then added to the reacting solution to reduce any unreacted aldehyde groups. Subsequent purifications *via* SEC and lyophilization resulted in purified EC-SEAL.

### Glass Coverslip Functionalization

Circular glass coverslips (25 mm diameter) were cleaned in piranha solution (1:1 mixture of sulfuric acid and 30% hydrogen peroxide) for 20 min. They were then rinsed in water and acetone and placed in a solution containing 2% 3-aminopropyltriethoxysilane (APTES, Thermo Fisher Scientific) in acetone for 5 min to create uniform aminosilane groups on the surface. Following a final rinse in acetone, coverslips were allowed to dry completely. To covalently attach Protein A/G (Thermo Fisher Scientific) to the surface, a solution containing 0.2 mg/mL Protein A/G and 0.25 mM of cross-linker bis(sulfosuccinimidyl)suberate (BS3, Thermo Fisher Scientific) was added to the coverslip and spread uniformly across the surface. Coverslips were then incubated overnight at 4 °C. The reaction was quenched with 1 mM Tris–hydrochloride (Tris–HCl, Corning, Inc., Corning, NY) for 15 min and coverslips were rinsed twice in 1 × PBS. 5 µg/mL of recombinant human E-selectin Fc chimera protein (R&D Systems, Minneapolis, MN), recombinant human P-selectin Fc chimera protein (R&D Systems) or recombinant human ICAM-1 Fc chimera protein (R&D Systems) was then distributed evenly across the surface and reacted at room temperature for a minimum of 2 h. Coverslips were then rinsed twice in 1 × PBS prior to use in subsequent experiments.

### Microfluidic Devices

Customized microfluidic devices were fabricated and assembled as previously described.^10^,^20^ Briefly, AutoCAD (Autodesk, San Rafael, CA) was used to design microfluidic channels and photolithography was utilized to pattern the design onto silicon wafers. Polydimethylsiloxane (PDMS) chips were produced by curing Sylgard^®^ 184 polymer (Dow Corning, Midland, MI) over the silicon wafer. After removal from the silicon wafer, access to flow chambers and vacuum ports was obtained by puncturing holes in the PDMS. The PDMS chip was then vacuum-sealed to a functionalized glass coverslip and 20-gauge needles were inserted for use as inlet reservoirs while a pump line was attached to the outlet to regulate flow. A syringe pump then applies negative pressure to create flow at desired shear rates in the multiple independent channels within each mold.

### EC-SEAL Binding to E-Selectin, P-Selectin, ICAM-1 and Neutrophils

Glass coverslips were coated with E-selectin, P-selectin or ICAM-1, blocked with 0.1% human serum albumin (HSA) in Hank’s balanced salt solution (HBSS) for 30 min and assembled on microfluidic devices as described above. Fluorescently-labeled EC-SEAL was suspended in 0.1% HSA in HBSS with Ca^2+^ and Mg^2+^ (Corning) to create the following treatment concentrations: 3, 10, 20, 30 and 60 *µ*M. Each treatment was added to an inlet reservoir and 0.1 dynes/cm^2^ of negative pressure was applied to draw them into the flow chambers. EC-SEAL treatments were incubated for 1 h and then the chambers were rinsed with 0.1% HSA in HBSS (again by applying negative pressure at 0.1 dynes/cm^2^). Fluorescent images were obtained using a Nikon TE2000 inverted microscope (Nikon, Minato, Tokyo, Japan) with a Plan Fluor 20X objective and mercury lamp with appropriate filter set. Images were captured with a 16-bit digital complementary metal–oxide semiconductor Andor Zyla camera (Andor, Belfast, Ireland) and Nikon Instruments Software (NIS) Elements imaging software (Nikon). For each individual chamber, five images were acquired in random locations and fluorescence was quantified using ImageJ (National Institutes of Health, Bethesda, MD). Background fluorescence of each coverslip (i.e., no EC-SEAL treatment) and EC-SEAL binding to Protein A/G coated surfaces (i.e. no adhesion molecule) were subtracted from fluorescent readings for each concentration to obtain final values. To observe EC-SEAL binding to neutrophils, fluorescently-labeled EC-SEAL (CF633 + biotin-conjugated EC-SEAL) was suspended in 0.1% HSA in HBSS with calcium and serially diluted to create the following treatment concentrations: 3, 10, 20, and 30 *µ*M. Isolated human neutrophils were treated with an M1/70 Mac-1 blocking antibody for 20 min in the dark on ice. Treated neutrophils were then centrifuged at 1500 × *g* for 5 min, supernatant was removed, and resuspended in serially diluted EC-SEAL for 1 h, at room temperature, in the dark, and with rotation. Samples were fixed with BioLegend Fixation buffer for 20 min, and run on the Attune NxT flow cytometer. Data was analyzed using FlowJo flow cytometry analysis software. All treatments were performed in duplicate.

### Endothelial Cell Cultures

Human dermal microvascular ECs (HMVECs, Lonza, Basel, Switzerland) were cultured in endothelial growth medium (EGM™-2, Lonza) with microvascular BulletKit™ supplement (Lonza). Cells were maintained at 37 °C and 5% CO_2_, and media for all cultures was changed every other day. Passage 3–7 ECs were seeded at a density of 1 × 10^5^ cells/cm^2^ in their respective media on tissue culture polystyrene and allowed to adhere for 24 h prior to any stimulation or treatment. For seeding HMVECs on glass coverslips, the circular coverslips (25 mm diameter) were autoclaved, placed in individual petri dishes and coated with 100 *µ*g/mL collagen type I (Corning) in 1 × PBS. After 1 h at room temperature, coverslips were rinsed with 1 × PBS. 200 *µ*L of HMVECs at 500,000 cells/mL was then added and distributed evenly across the surface of the coverslip. HMVECs were allowed to adhere for at least 20 min prior to additional media being added and subsequent incubation at 37 °C and 5% CO_2_.

### Endothelial Cell Characterization

HMVEC monolayers were seeded on 12-well tissue culture plates (Corning) and treated with either control (unstimulated) media or 0.3 ng/mL TNF-*α* + 0.2 ng/mL IL-1*β* for 4 h. HMVECs were rinsed with 1 × PBS and detached from the tissue culture plate with 0.25% trypsin (Lonza). HMVECs were then centrifuged at 1500 × *g* and 4 °C and resuspended in human TruStain FcX blocking solution (BioLegend, San Diego, CA) for 20 min on ice. The following antibodies were then added to HMVECs in separate vials: FITC conjugated mouse anti-human P-selectin (BioLegend), PE/Cy5 conjugated mouse anti-human ICAM-1 (BioLegend), PE conjugated mouse anti-human VCAM-1 (BioLegend), PE conjugated mouse anti-human E-selectin (BioLegend). After a brief (< 1 s) vortex and incubation on ice for 30 min, HMVECs were rinsed, centrifuged (1500 × *g*, 4 °C) and resuspended in 0.2% HSA in HBSS. Samples were then run on the Attune NxT flow cytometer (Thermo Fisher Scientific). A separate vial stained with 7-aminoactinomycin (7-AAD) was also run at the end of the experiment to determine cell viability. Data was analyzed using the software program FlowJo (FlowJo, LLC, Ashland, OR).

### Neutrophil Isolation

Neutrophils were isolated as described previously.^2^ Briefly, whole blood was obtained from healthy volunteers in collection vials containing sodium citrate. 5 mL of neutrophil isolation media (Polymorphprep™, Cosmo Bio USA, Inc., Carlsbad, CA) was added to the bottom of a 15 mL conical tube and 5 mL of whole blood was gently pipetted on top. After centrifuging at 760 × *g* for 30 min at room temperature, the neutrophil layer was collected using a pipette and placed in a 50 mL conical tube containing 30 mL of 0.1% HSA in HBSS (without Ca^2+^). Neutrophils were then centrifuged for 8 min at 190 × *g* and room temperature. Isolated neutrophils were resuspended in 1 mL of new 0.1% HSA in HBSS (without Ca^2+^). Cell counts were obtained using a Coulter counter and neutrophils were suspended to a concentration of 1 × 10^6^ cells/mL. Finally, they were placed on a rocker for gentle rolling until transfer to Ca^2+^ containing HBSS for use in experiments.

Neutrophils were isolated from freshly collected human blood from healthy donors consented through an approved University of California, Davis Institutional Review Board Protocol #235586-11.

### Bead Functionalization

E- and P-selectin coated beads were fabricated as follows: Polybead^®^ amine-modified fluorescent microspheres (AF647, 6 *µ*m diameter, 2.5% w/v) and associated reagents were purchased from Polysciences, Inc. (Warrington, PA). These microspheres (or beads) were rinsed and reacted with 8% glutaraldehyde in 1 × PBS at room temperature overnight to create aldehyde groups on the bead surface. The beads were then centrifuged for 15 min at 1200 × *g* and the supernatant was removed. The pellet was resuspended in 1 × PBS and rinsed twice. 10 *µ*g/mL of recombinant human E-selectin or P-selectin Fc chimera protein was added to the beads and incubated for 4 h with gentle mixing throughout. The beads were centrifuged again at 1200 × *g* for 15 min, the supernatant was removed and the reaction was quenched with 500 mM ethanolamine for 30 min. Following another round of centrifugation, the pellet of beads was resuspended in 1% casein for 30 min to block non-specific interactions. After two more centrifugation/rinse cycles in 1% casein, beads were stored at 4 °C in storage buffer (1% casein containing 0.1% sodium azide and 5% glycerol) for use in future experiments.

### Neutrophil Binding to Beads

E- or P-selectin coated beads were incubated with EC-SEAL (with or without calcium present) or the selectin-antagonist small molecule GMI-1070^6^ (GlycoMimetics, Inc., Rockville, MD) for 1 h at room temperature. Isolated neutrophils were labeled with PE anti-human CD16 [3G8] (BioLegend) to characterize them as neutrophils and Alexa Fluor 488 anti-human CD11a/CD18 (LFA-1) (BioLegend) to assess level of neutrophil activation. Treated beads were then centrifuged at 1000 × *g* for 3 min, supernatant was removed, and labeled neutrophils were added. Following 15 min of incubation at 4 °C with shaking, samples were run on the Attune NxT flow cytometer. Bead population and neutrophil population were identified based upon their characteristic FSC and SSC profiles. The bead population was identified by AF647 fluorescence intensity to discriminate singlet, doublet, and triplet populations based upon MFI of single beads. Neutrophil populations were identified by fluorescent PE anti-CD16 antibody, and quantitation of bead-cell was determined based upon the combination of AF647 and PE fluorescence intensity. To confirm the presence of E- or P-selectin on the surface of the beads, a separate experiment was performed using beads treated with PE mouse anti-human E-selectin antibody or FITC mouse anti-human P-selectin antibody, respectively. Flow cytometry data was analyzed using FlowJo.

### Neutrophil Rolling–Arrest–Migration

Coverslips were prepared with E-selectin alone, a combination of E-selectin and ICAM-1 or HMVECs as described above and blocked with 0.1% HSA in HBSS. Functionalized and blocked coverslips were treated with vehicle control (no treatment), 30 *µ*M EC-SEAL or 6.5 *µ*M GMI-1070 for 1 h, rinsed with 0.1% HSA in HBSS and assembled on microfluidic devices. Isolated neutrophils (5 × 10^5^ cells/mL) were placed into the inlet reservoirs and flow was initiated to create a shear stress of 2 dynes/cm^2^. Three minutes after pump initialization, a Nikon TE2000 inverted microscope with accompanying NIS Elements software was utilized to capture phase contrast images of neutrophils interacting with the substrate. Images were taken in the middle of each channel every 3 s for 10 min. Following image acquisition, the ImageJ plugin MTrack2 was used to track positions of rolling neutrophils which in turn, provided for the calculation of rolling velocities. Percentage of rolling neutrophils transitioning to arrest and subsequent migration were calculated for the combination E-selectin/ICAM-1 substrate as well as HMVECs.

### Statistics

All experiments were performed in triplicate or quadruplicate and results are presented as mean ± standard deviation, unless otherwise noted. GraphPad Prism software (GraphPad Software, Inc., La Jolla, CA) was used to perform statistical analysis. All results were analyzed using ANOVA with post hoc Tukey test. Statistical significance threshold was set at *p* < 0.05.

## Results

### EC-SEAL Binding to E-Selectin, P-Selectin and ICAM-1

**Figure 1:**
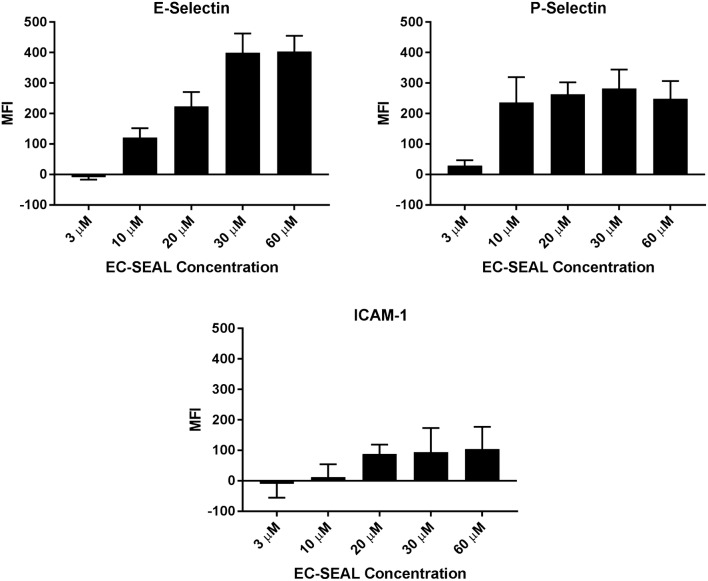
EC-SEAL binding to E-selectin, P-selectin and ICAM-1. Binding of labeled EC-SEAL to adhesion molecules was quantified using fluorescence microscopy (mean fluorescence intensity, MFI). Binding to each adhesion molecule was detected at each concentration and non-specific background was subtracted. *n* = 3; *p* < 0.05.

To measure the capacity of EC-SEAL to specifically target endothelial adhesion molecules, a fluorescent tag (CF633) was added during EC-SEAL synthesis to allow for direct visualization under fluorescent microscopy. Functionalized glass coverslips were coated with recombinant E-selectin, P-selectin or ICAM-1 and treated with varying concentrations of EC-SEAL. As shown in Fig. 1, EC-SEAL exhibited the capacity to bind all three adhesion molecules examined with a hierarchy of affinity E-selectin > P-selectin > ICAM-1. This trend is consistent with previous work regarding the targeting of IEL peptide to E- and P-selectin.^9^ Additionally, EC-SEAL was shown to bind directly to neutrophils, presumably through interaction with L-selectin (Supplemental Fig. 1).

### Neutrophil Binding to Beads

**Figure 2:**
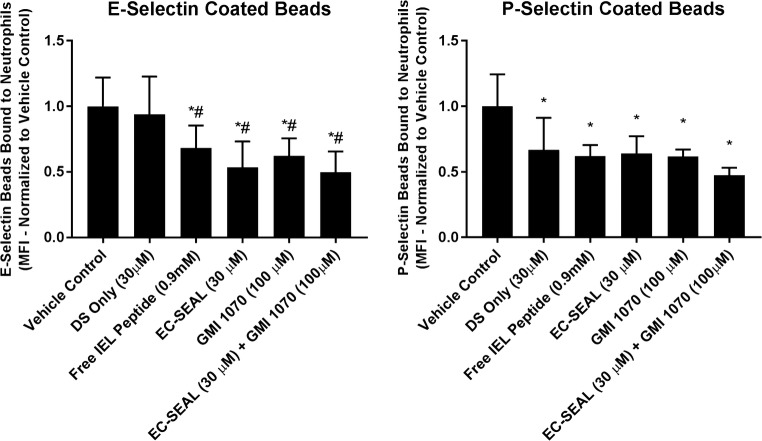
Neutrophil binding to E- and P-selectin coated beads. Fluorescent beads coated with E- or P-selectin were treated with free IEL peptide, DS only, EC-SEAL, GMI-1070 or combined EC-SEAL/GMI-1070 and incubated with neutrophils (concentration = 10^6^/mL). Neutrophil-bead binding was assessed using flow cytometry and all values were normalized to vehicle control (mean fluorescence intensity, MFI). *Represents a significant difference from the vehicle control, ^#^represents a significant difference from DS only (30 *µ*M). *n* = 6 (E-selectin), *n* = 3 (P-selectin), *p* < 0.05.

To investigate the relative capacity of EC-SEAL to inhibit neutrophil interactions through its selectin-binding action, E- or P-selectin coated fluorescent beads were treated with EC-SEAL and/or the selectin-antagonist GMI-1070. Neutrophils were then added and neutrophil-bead binding in shear mixed suspensions were quantified using flow cytometry. Free IEL peptide (0.9 mM, at molar equivalent to that conjugated to EC-SEAL, 30 *µ*M), EC-SEAL (30 *µ*M), GMI-1070 (100 *µ*M) and combined EC-SEAL/GMI-1070 (30/100 *µ*M) decreased neutrophil binding to either E- or P-selectin coated beads by 35–50% compared to vehicle control (Fig. 2). DS only (30 *µ*M) had no effect on neutrophil binding to E-selectin coated beads, but did decrease neutrophil interactions with P-selectin coated beads at a level similar to other treatments. This difference suggests that DS plays a role in EC-SEAL binding to P-selectin. Combining EC-SEAL and GMI-1070 did not exert additive inhibition, suggesting that the two molecules share a similar mechanism in antagonism of neutrophil binding. Additionally, EC-SEAL did not reduce neutrophil binding to E-selectin coated beads without calcium present in the media (Supplemental Fig. 2), indicating the calcium-dependent nature of the EC-SEAL/E-selectin interaction.

### Neutrophil Rolling on E-Selectin

**Figure 3:**
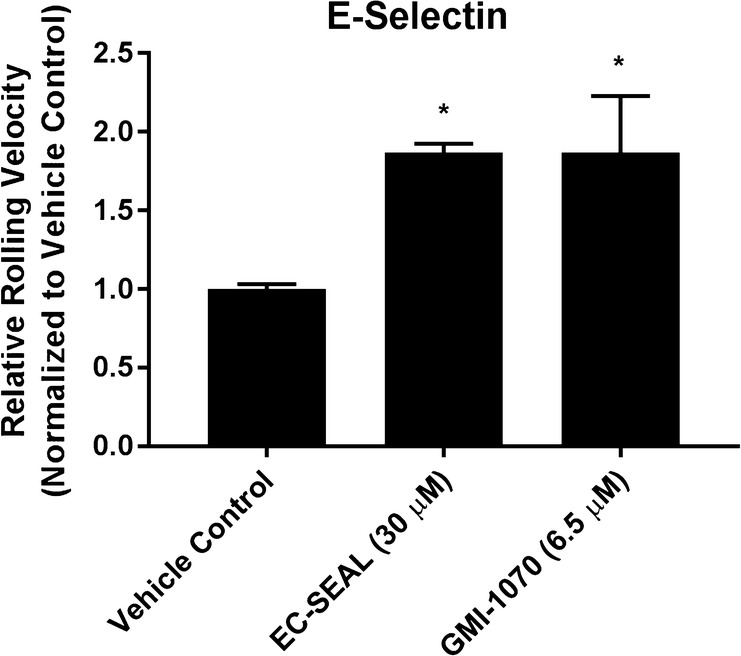
Neutrophil rolling velocities on E-selectin. Neutrophils were added to the inlet reservoir of a microfluidic device and a syringe pump applied negative pressure at the outlet to create a shear stress of 2 dynes/cm^2^ in the flow chamber. Neutrophils rolling on the substrate surface had their positions tracked, and rolling velocities were calculated by determining the distance traveled and dividing by the time each neutrophil was in the field of view. Results were normalized to vehicle control (untreated) for analysis. EC-SEAL (30 *µ*M) and GMI-1070 (6.5 *µ*M) caused an approximately 2-fold increase in rolling velocity. *Represents a significant difference from the vehicle control. *n* = 4, *p* < 0.05.

To quantify the effect of EC-SEAL on selectin mediated capture and rolling under shear flow, neutrophils were sheared on coverslips coated with E-selectin with various treatment conditions (EC-SEAL, GMI-1070 or vehicle control). As shown in Fig. 3, 30 *µ*M EC-SEAL and 6.5 *µ*M GMI-1070 increased neutrophil rolling velocity on E-selectin by approximately 2-fold compared to vehicle control.

### Neutrophil Rolling–Arrest–Migration on E-Selectin/ICAM-1 and HMVECs

**Figure 4:**
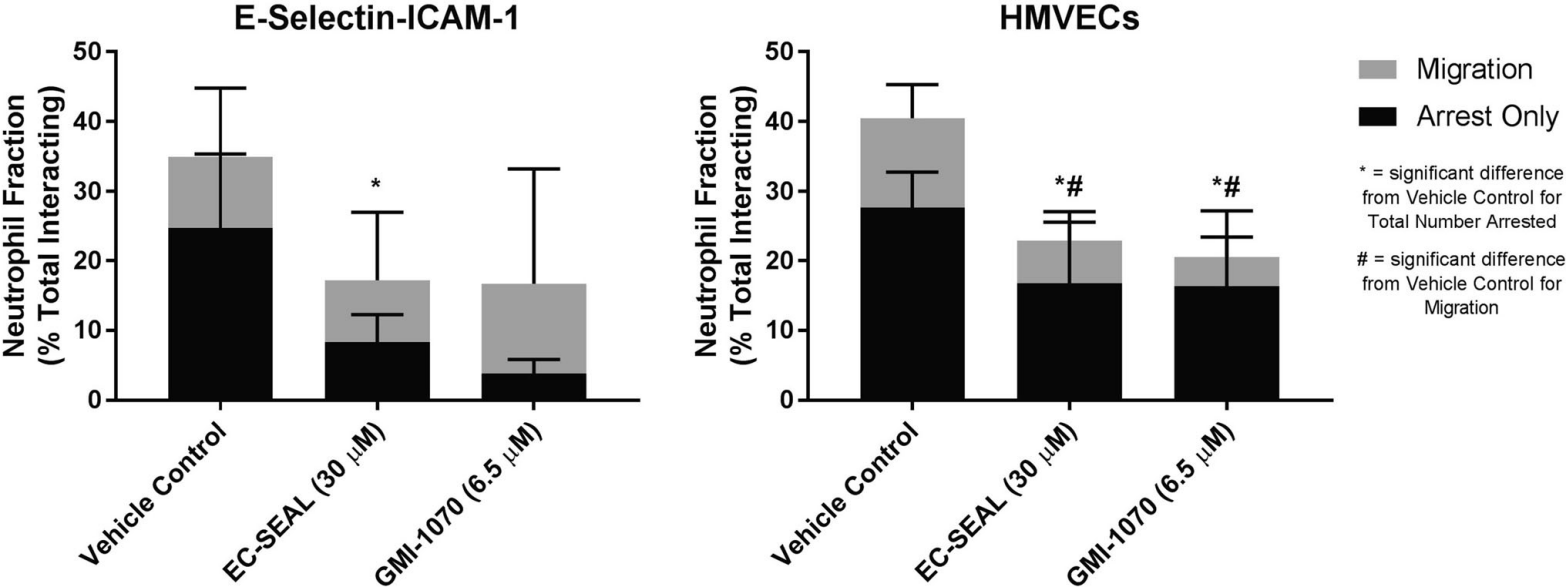
Neutrophil transition from rolling to arrest on E-selectin/ICAM-1 and HMVECs. E-selectin/ICAM-1 substrates or inflamed HMVECs were treated with EC-SEAL (30 *µ*M) or GMI-1070 (6.5 *µ*M). Neutrophils were added to the inlet reservoir of a microfluidic device and a syringe pump applied negative pressure at the outlet to create a shear stress of 2 dynes/cm^2^ in the flow chamber. Positional tracking and manual counts allowed for distinguishing between rolling vs. arrested vs. migrated neutrophils. On E-selectin/ICAM-1 substrates, EC-SEAL significantly reduced neutrophil transition to arrest compared to vehicle control, while neither treatment reduced migration of arrested neutrophils. On inflamed HMVECs, both EC-SEAL and GMI-1070 reduced neutrophil transition to arrest and subsequent migration. *n* = 4, *p* < 0.05.

The transition from neutrophil rolling to arrest and subsequent migration requires the binding to ICAM-1 on the inflamed EC membrane. Therefore, studies were undertaken to determine EC-SEAL’s ability to antagonize neutrophil transition to arrest under shear flow in microfluidic channels. To examine rolling, arrest and migration on ICAM-1 plus E-selectin surfaces, we coated the substrate with a combination of E-selectin/ICAM-1. EC-SEAL decreased the number of rolling neutrophils transitioning to arrest by approximately 50% compared to vehicle control (Fig. 4). GMI-1070 did not significantly reduce this transition, but it trended to decrease arrest (*p* = 0.12). Neither EC-SEAL nor GMI-1070 decreased neutrophil migration following arrest on E-selectin/ICAM-1.

We next examined neutrophil rolling and arrest on inflamed HMVECs. HMVECs were probed for baseline expression of E-selectin, P-selectin and ICAM-1 with and without IL-1*β* and TNF-*α* stimulation using antibodies to the respective receptors. Results showed that P-selectin is constitutively expressed at low levels and its cell surface presentation is unchanged 4 h following stimulation, while E-selectin presentation is increased ~ 3-fold and ICAM presentation increased ~ 7-fold with stimulation. Neutrophil transition from rolling to arrest was then examined on HMVECs that were stimulated with TNF-*α* and IL-1*β* prior to EC-SEAL and GMI-1070 treatments. Compared to vehicle control, EC-SEAL (30 *µ*M) and GMI-1070 (6.5 *µ*M) both significantly decreased the total number of neutrophils transitioning from rolling to arrest (Fig. 4). Additionally, both treatments reduced neutrophil transmigration on the HMVEC monolayer.

## Discussion

Of the many aspects associated with endothelial dysfunction, neutrophils play a pivotal role.^5^,^8^,^12^,^13^ From the production of reactive oxygen species at the site of inflammation, to the response to and production of proinflammatory cytokines and chemokines, and the increased interactions with upregulated endothelial adhesion molecules, neutrophils are implicated in each insult.^12^,^13^ Chronic inflammation in endothelial dysfunction has been linked to cardiovascular disease and currently no consistent, viable treatment is available.^18^ In the current study, we examined the capacity of a selectin-targeting glycocalyx mimetic (termed EC-SEAL) designed to bind to adhesion receptors upregulated on the EC surface, to reduce neutrophil recruitment in the inflamed state.

The capacity of EC-SEAL to target EC adhesion molecules was examined using amine-functionalized glass substrates presenting recombinant E-selectin, P-selectin or ICAM-1 conjugated to the surface. These studies demonstrated that EC-SEAL binds significantly tighter to selectins as compared to ICAM-1. This finding is consistent with the design of EC-SEAL in which multiple selectin-binding peptides are displayed on its glycosaminoglycan backbone. Further, EC-SEAL exhibited greater binding to E-selectin compared to P-selectin. It has been shown previously that the specific peptide sequence used in EC-SEAL [IELLQARGC (IEL)] targets both E- and P-selectin, but has a higher affinity for E-selectin.^9^ Although not designed to specifically target ICAM-1, a significant level of EC-SEAL binding to ICAM-1 was detected at concentrations of 20 *µ*M and greater. This interaction may be electrostatic in nature given the high net negative charge of DS, or may be some nonspecific interaction between the peptide and ICAM-1. EC-SEAL was also shown to bind directly to neutrophils, presumably through interaction with L-selectin which is constitutively expressed on the neutrophil surface. However, EC-SEAL binding to neutrophils does not appear to increase interactions of neutrophils to E-selectin surfaces or to activated endothelium as evidenced by the increased neutrophil rolling velocity and decreased neutrophil arrest and migration on activated endothelium in the presence of EC-SEAL. The ability of EC-SEAL to preferentially target selectins while maintaining some cross-reactivity with ICAM-1 is viewed as advantageous given the sequential nature of neutrophil rolling to arrest on endothelium. It has been reported that the glycocalyx provides a steric and electrostatic layer that can prevent cells from binding their cognate counter-receptors. For example, Schmidt, *et al*. have shown that the endothelial glycocalyx regulates neutrophil adhesion and tissue invasion during sepsis as a result of glycocalyx degradation and adhesion receptor exposure.^22^

In order to confirm expression of adhesion receptors upregulated on the endothelial surface, ECs were characterized using flow cytometry. Stimulation with TNF-*α* and IL-1*β* resulted in a dynamic increase in the expression of E-selectin and ICAM-1. P-selectin, however, was not upregulated at 4 h after stimulation. This finding is likely due the fact that P-selectin is preformed and stored in Weibel–Palade bodies which are released within minutes of EC activation, a time point that was not examined in this study.^4^,^12^ Alternatively, E-selectin is produced *de novo* and takes approximately 90 min before its membrane protein concentration increases.^12^ Therefore, it is possible that when measuring adhesion molecule expression at 4 h after stimulation, early P-selectin upregulation was missed, while the later increase in E-selectin membrane concentration was captured.

To investigate the reduction in neutrophil interactions discretely *via* E- vs. P-selectin mediated mechanisms, the ability of EC-SEAL to prevent neutrophil binding to E- and P-selectin coated beads in a mixed suspension was examined. All treatment conditions with the exception of those lacking calcium and DS only inhibited neutrophil binding to both E- and P-selectin coated beads compared to vehicle control. It is noteworthy that the DS only treatment condition decreased neutrophil binding to P-selectin coated beads, but not E-selectin. E- and P-selectin are very similar structurally, although P-selectin contains a greater number of cysteine rich repeating units.^7^ It is proposed that the binding of DS takes place in the region with increased length in P-selectin. This explanation may offer additional insight into the EC-SEAL binding results. The binding of EC-SEAL to P-selectin saturates at a lower concentration compared with E-selectin, likely through the increased interaction of DS with the longer region of cysteine rich repeating units in P-selectin. However, EC-SEAL ultimately exhibited greater binding that was dose dependent to E-selectin with increased EC-SEAL concentrations, driven by the IEL peptide–E-selectin interactions. GMI-1070 is a sialyl Lewis-x tetrasacharide mimetic that functions as a selectin-antagonist^6^ and served as a positive control in the neutrophil-bead binding experiments. As anticipated, the combination treatment of EC-SEAL + GMI-1070 did not show enhanced inhibition of neutrophil binding, indicating that the location of selectin-binding or mechanism of antagonism is similar for the two molecules.

In order to investigate the effectiveness of EC-SEAL on neutrophil interactions under shear stress as is present in blood vessels, experiments observing these interactions under flow were conducted. Specifically, microfluidic devices were utilized to examine neutrophil rolling velocities on E-selectin coated substrates. EC-SEAL caused rolling velocity to double on average, compared to vehicle control (Fig. 3). This increase in rolling velocity is due to significant inhibition of E-selectin recognition of its respective ligands on neutrophils. With fewer selectin sites for tethered attachment available, neutrophils form fewer tethered bonds as they travel a greater distance between possible bonding sites. Ultimately, the key event determining if a neutrophil migrates to an inflammatory site in tissue is whether or not the neutrophil arrests on the EC surface.^12^ The ability of EC-SEAL to affect the transition to arrest was observed on E-selectin/ICAM-1 coated substrates and inflamed HMVECs. EC-SEAL decreased the percentage of rolling neutrophils transitioning to arrest compared to vehicle control on E-selectin/ICAM-1 substrates, as well as inflamed HMVECs (Fig. 4). EC-SEAL also reduced the percentage of arrested cells that transmigrated through the HMVECs. The proposed mechanism for the inhibitory capacity of EC-SEAL is 2-fold: (1) blocking E- and P-selectin decreases neutrophil interactions and increases rolling velocity and (2) directly binding ICAM-1 (and potentially other integrins) inhibits the ability of neutrophils to transition to integrin mediated arrest. This inhibition of neutrophil rolling to arrest in the presence of EC-SEAL was corroborated using GMI-1070 (6.5 *µ*M). As mentioned previously, GMI-1070 is a known selectin antagonist with a preference for E-selectin.^6^ This molecule has been shown to reduce selectin-mediated neutrophil rolling and specifically, 6.5 *µ*M is known to be an effective, yet non-saturating dose.^6^ Importantly, administering the proinflammatory cytokine IL-8 at the end of an experiment caused near immediate arrest of all neutrophils regardless of previous treatment applied. This indicates that the “inside-out” mechanism of activation for neutrophils in which *β*_2_-integrins become allosterically activated following a stimulus remains intact. It is also further proof that EC-SEAL is inhibiting neutrophil arrest on the EC surface by disrupting the “outside-in” pathway by which neutrophil rolling causes L-selectin receptor ligand clustering that signals integrin mediated shear resistant arrest.

## Conclusions

The use of a novel selectin-targeting glycocalyx mimetic (termed EC-SEAL) to inhibit leukocyte interactions associated with endothelial inflammation was investigated. EC-SEAL was shown to preferentially bind to E- and P-selectin, and also displayed weaker binding to ICAM-1. Under physiological levels of shear stress, EC-SEAL increased the rolling velocity of neutrophils on E-selectin substrates, and decreased the transition to arrest on E-selectin/ICAM-1 substrates and HMVECs. Overall, EC-SEAL shows promise as a potential therapeutic in the treatment of endothelial dysfunction and associated inflammatory disease states.

## Electronic supplementary material

Below is the link to the electronic supplementary material.

Supplementary material 1 (JPG 61 kb) **Supplemental Fig. 1**. EC-SEAL binding to neutrophils (L-selectin). Binding of labeled EC-SEAL to neutrophils was quantified using fluorescence microscopy (mean fluorescence intensity, MFI). Binding to neutrophils was detected at each concentration and non-specific background was subtracted. *n* = 3, *p* < 0.05.

Supplementary material 2 (JPG 53 kb) **Supplemental Fig. 2**. Neutrophil binding to E-selectin coated beads in the presence and absence of calcium. Fluorescent beads coated with E-selectin were treated with EC-SEAL (with and without calcium) and incubated with neutrophils (concentration = 106/mL). Neutrophil-bead binding was assessed using flow cytometry and all values were normalized to calcium control (mean fluorescence intensity, MFI). *Represents a significant difference from the calcium control. *n* = 3, *p* < 0.05.
