# Supplementary figures and images for: Selectin-Targeting Peptide–Glycosaminoglycan Conjugates Modulate Neutrophil–Endothelial Interactions
Source: Cell Mol Bioeng. 2018 Sep 17;12(1):121–30. doi: 10.1007/s12195-018-0555-6 (PMC6345733; doi:10.1007/s12195-018-0555-6)

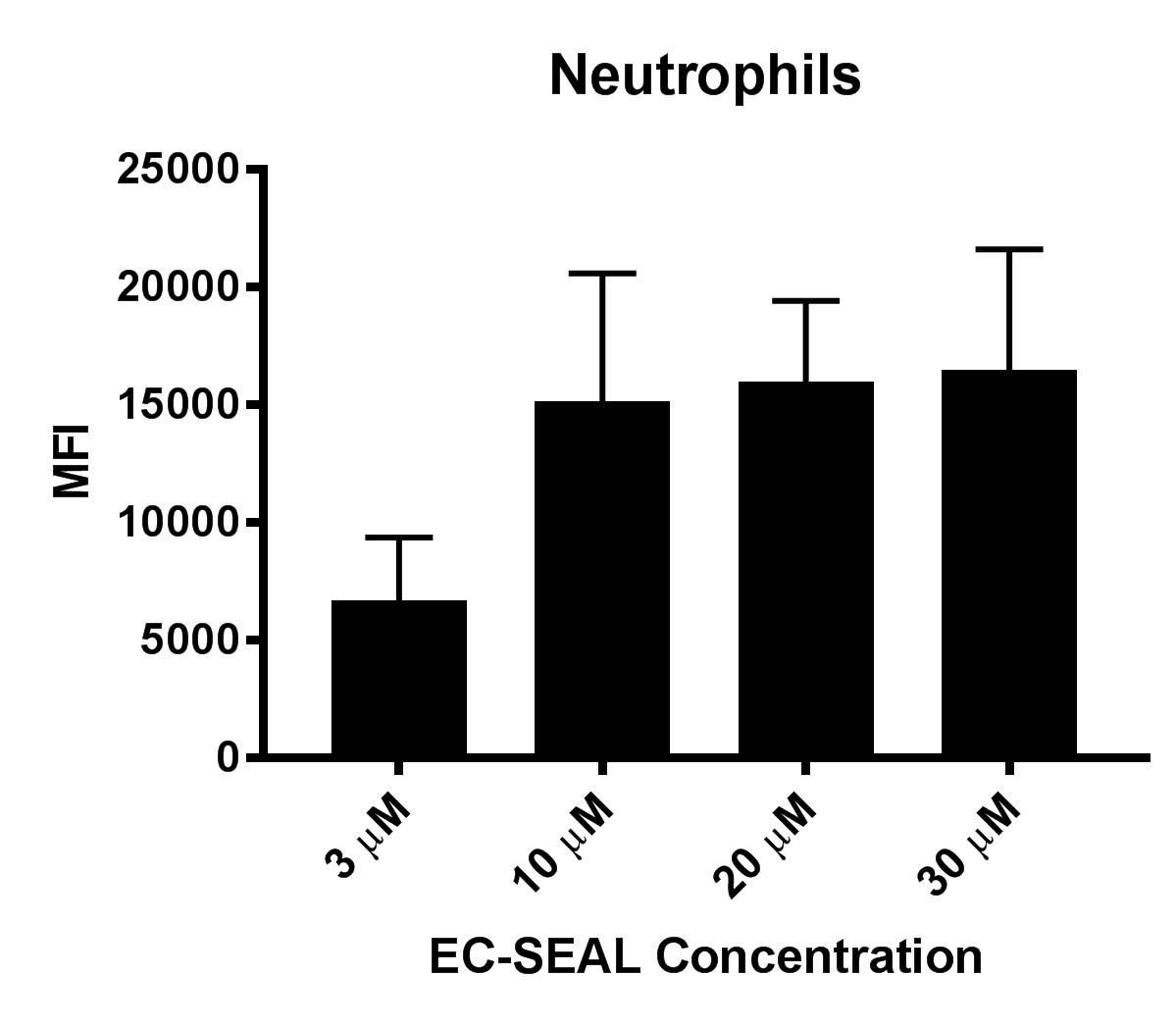

Supplement: Supplementary file 1 — Supplementary material 1 (JPG 61 kb) Supplemental Fig. 1. EC-SEAL binding to neutrophils (L-selectin). Binding of labeled EC-SEAL to neutrophils was quantified using fluorescence microscopy (mean fluorescence intensity, MFI). Binding to neutrophils was detected at each concentration and non-specific background was subtracted. n = 3, p < 0.05. [file 12195_2018_555_MOESM1_ESM.jpg]

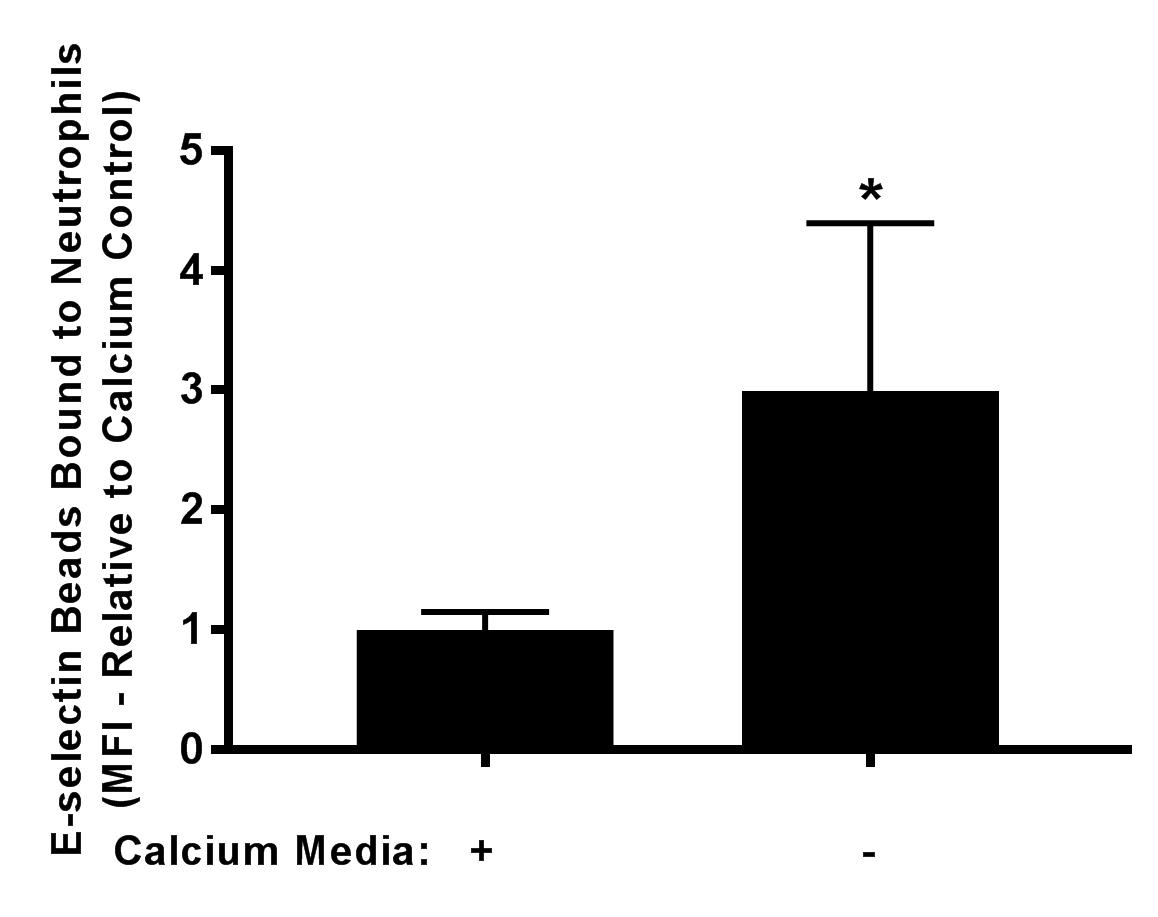

Supplement: Supplementary file 2 — Supplementary material 2 (JPG 53 kb) Supplemental Fig. 2. Neutrophil binding to E-selectin coated beads in the presence and absence of calcium. Fluorescent beads coated with E-selectin were treated with EC-SEAL (with and without calcium) and incubated with neutrophils (concentration = 106/mL). Neutrophil-bead binding was assessed using flow cytometry and all values were normalized to calcium control (mean fluorescence intensity, MFI). *Represents a significant difference from the calcium control. n = 3, p < 0.05. [file 12195_2018_555_MOESM2_ESM.jpg]
